# Effects of ^99m^Tc-TRODAT-1 drug template on image quantitative analysis

**DOI:** 10.1371/journal.pone.0194503

**Published:** 2018-03-15

**Authors:** Cheng-Han Wu, Bang-Hung Yang, Yuan-Hwa Chou, Shyh-Jen Wang, Jyh-Cheng Chen

**Affiliations:** 1 Department of Biomedical Imaging and Radiological Sciences, National Yang-Ming University, Taipei, Taiwan; 2 Department of Nuclear Medicine, Taipei Veterans General Hospital, Taipei, Taiwan; 3 Department of Psychiatry, Taipei Veterans General Hospital, Taipei, Taiwan; 4 Faculty of Medicine, National Yang-Ming University, Taipei, Taiwan; Waseda University, JAPAN

## Abstract

^99m^Tc-TRODAT-1 is a type of drug that can bind to dopamine transporters in living organisms and is often used in SPCT imaging for observation of changes in the activity uptake of dopamine in the striatum. Therefore, it is currently widely used in studies on clinical diagnosis of Parkinson’s disease (PD) and movement-related disorders. In conventional ^99m^Tc-TRODAT-1 SPECT image evaluation, visual inspection or manual selection of ROI for semiquantitative analysis is mainly used to observe and evaluate the degree of striatal defects. However, these methods are dependent on the subjective opinions of observers, which lead to human errors, have shortcomings such as long duration, increased effort, and have low reproducibility. To solve this problem, this study aimed to establish an automatic semiquantitative analytical method for ^99m^Tc-TRODAT-1. This method combines three drug templates (one built-in SPECT template in SPM software and two self-generated MRI-based and HMPAO-based TRODAT-1 templates) for the semiquantitative analysis of the striatal phantom and clinical images. At the same time, the results of automatic analysis of the three templates were compared with results from a conventional manual analysis for examining the feasibility of automatic analysis and the effects of drug templates on automatic semiquantitative analysis results. After comparison, it was found that the MRI-based TRODAT-1 template generated from MRI images is the most suitable template for ^99m^Tc-TRODAT-1 automatic semiquantitative analysis.

## Introduction

Dopamine transporters are located in the presynaptic cell membrane at dopaminergic neuronal endings and play an important role in regulating extracellular dopamine levels. Therefore, these transporters can affect the number of neural signals received by the striatum. Conditions affecting these transporters can thus cause Parkinson’s disease (PD) of varying severity. Thus, dopamine transporters could be used as a biomarker for evaluating striatal neuronal function [1, 2]. When ^99m^Tc is labeled on the tropane analog TRODAT-1, ^99m^Tc-TRODAT-1 is formed. This radiopharmaceutical or drug has been confirmed to bind to dopamine transporters in the living organisms [3]. Therefore, SPECT imaging can be used to make three-dimensional (3D) observations of changes in the radioactivity uptake in the striatum. Currently, this has been mainly used in studies on the clinical diagnosis of PD and movement-related disorders [4, 5].

In conventional SPECT image evaluation, visual inspection or manual selection of ROI for semiquantitative analysis is mainly used to evaluate the degree of striatal defects [4]. However, these methods are dependent on the subjective opinions of observers, which lead to human errors, and have shortcomings such as long duration, increased effort, and low reproducibility [6, 7]. Therefore, to solve these problems, many automatic semiquantitative analytical methods and software have been created. Examples include the use of QuantiSPECT (GE Healthcare, Amersham, Buckinghamshire, UK) for DaTSCAN images in ^123^I-FP-CIT SPECT imaging. This software provides three semiquantitative analytical methods, including the two-box method proposed by Fleming and Bolt et al. [8], the three-box method proposed by Costa et al. [9], and the crescent method proposed by Lokkegaard et al. [10]. These three methods can automatically select ROI directly in ^123^I-FP-CIT SPECT images for semiquantitative analysis. The study by Morton et al. compared these three methods in detail [11, 12]. Apart from these methods, the IBZM-tool developed by Buchert et al. [7] using statistical parametric mapping (SPM) software employs another type of semiquantitative analytical method. This method is dependent on the ^123^I-IBZM SPECT template that conforms to the Montreal Neurological Institute space (MNI space) as well as predefined ROI that similarly conforms to MNI space, and is different from the abovementioned methods. This is because all images must first undergo spatial normalization with the template, after which ROI is applied to all normalized images for semiquantitative analysis. Therefore, the drug templates play an important role in the accuracy of this analytical method.

In this study, we compared three drug templates with the aim of establishing an automatic semiquantitative analytical method exclusively for ^99m^Tc-TRODAT-1. Among these templates, two were ^99m^Tc-TRODAT-1 templates created in this study, whereas another was the SPECT template that is built-in in SPM. The three templates are all used in striatal phantoms and clinical images for semiquantitative analysis. At the same time, the results of semiquantitative analysis using these three methods were compared with the results from conventional manual analysis to identify the optimal ^99m^Tc-TRODAT-1 template. We hope that this template can be used in automatic semiquantitative analysis of ^99m^Tc-TRODAT-1 SPECT images to quantify the specific uptake ratio (SUR) in images of patients with PD. In addition, we hope that this template could improve the problems associated with conventional manual selection methods such as long duration, increased effort, low reproducibility, and human subjectivity.

## Material and methods

### SPECT imaging procedure

This study collected images from a total of 142 subjects, including 58 healthy subjects (25 males, 33 females, average age 26.55 ± 7.72 years) and 84 PD patients (42 males and 42 females, average age 69.44 ± 11.6 years). The fan-beam collimator in E.CAM (Siemens Medical Solutions USA, Inc.) was used for imaging. Every patient was administered 740 MBq (20 mCi) of ^99m^Tc-TRODAT-1 by intravenous injection and imaging was performed approximately 4 hours after injection [3, 13]. During imaging, the patients were in the supine position on an examination table with a fixed head frame. Symmetrical windows with ^99m^Tc energy peaks of 140 keV ± 10% were used, and the step-and-shoot scan mode was used with 360° rotation in the clockwise direction, obtaining projections at intervals of 3°. Each projection data was collected for 20 seconds, for a total of 120 projections. The image reconstruction matrix size was 128 × 128, with every pixel size and slice thickness being 3.89 mm. The Metz filter was used for filtered backprojection (FBP) reconstruction; the cutoff frequency used for the filter was 0.55 times the Nyquist frequency and the power order was 30. First-order Chang’s method was used for attenuation correction and the attenuation coefficient (μ) was 0.12 cm^−1^ [4, 14]. This study was approved by the institutional review board of Taipei Veterans General Hospital, and written informed consent was obtained for all subjects.

### Radiopharmaceutical

This experiment primarily used the ^99m^Tc-TRODAT-1 drug for imaging of dopamine transporters. The TRODAT-1 drug was obtained from the lyophilized vials of dopamine transporter imaging reagents from the Institute of Nuclear Energy Research at Lungtan, Taiwan. Five milliliters of physiological saline containing 1480 MBq of ^99m^Tc sodium pertechnetate (NaTcO_4_) was injected into the lyophilized vials and placed in a lead container for shaking until the contents have completely dissolved. The container was then placed in a high-temperature steam sterilizer and heated for 30 min at 121°C. After cooling to room temperature, the radioactivity and chemical purity were confirmed to be 99% [4].

### Manual ROI method

Three transverse slices of the reconstructed ^99m^Tc-TRODAT-1 images with the greatest activity uptake were selected and summed to form an image. ROI of the striatum and occipital lobe were manually selected on this image by experienced radiology technicians [4], and the average values of pixels in individual ROI were calculated. The striatum was then used as a target region and the occipital lobe was used as the background region for semiquantitative analysis.

### Automatic ROI method

**Fig 1:**
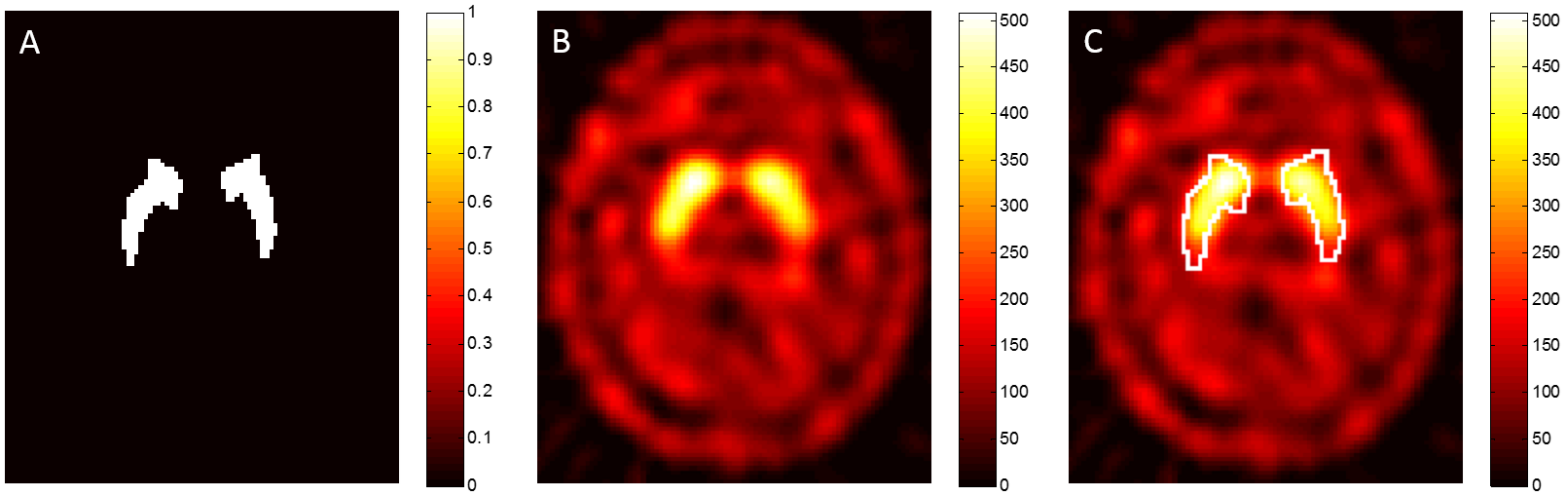
(A) Using the caudate and putamen regions in the AAL ROI template to define the striatal region, (B) ^99m^Tc-TRODAT-1 images that have undergone spatial normalization, (C) Using (A) to perform automatic striatal selection in ^99m^Tc-TRODAT-1 images.

In this study, our custom-made software was used for fully automatic ROI selection of images. This software was programmed using MATLAB (MathWorks Inc., Sherborn, MA), and it could perform analysis and evaluation of ^99m^Tc-TRODAT-1 images. The “Normalise” module of the SPM8 software (http://www.fil.ion.ucl.ac.uk/spm/software/) was used in our custom-made software [15, 16]. Spatial normalization of all ^99m^Tc-TRODAT-1 images was first performed using a standard MNI space brain template. The minimized mean-squared difference between the source image and drug template was calculated so that the spatial coordinates of the source image and the size of voxels were consistent with the template in standard MNI space [7, 17, 18]. Apart from modifying the bounding box to [−90 −126 −72; 90 90 108] so that the image after normalization can reflect the template in the standard MNI brain space, other spatial normalization parameters were all adopted from the built-in settings in SPM8, i.e. the 12-parameter affine transformation was used to represent changes in image translation, zooming, rotation, and shearing, 7 × 9 × 7 discrete cosine transform basis function, and no weighting was performed in the source image and template image. The cutoff of the discrete cosine transformation was 25 mm, and the nonlinear regularization term in 16 nonlinear iterations was set as 1. Modulation (meaning “preserve concentrations”) was not used. Following that, trilinear interpolation method was used for image reslicing. After normalization, the voxel size of the image was 2 × 2 × 2 mm^3^. Finally, the region of caudate, putamen, and occipital lobe defined in the automated anatomical labeling (AAL) ROI template that also conformed to MNI spatial coordinate axes was used for automatic ROI selection of normalized ^99m^Tc-TRODAT-1 images (Fig 1). Similarly, the striatum (caudate plus putamen regions) was used as the target region, and the occipital lobe was used as the background region for semiquantitative analysis [19].

### Creation of the ^99m^Tc-TRODAT-1 template

**Fig 2:**
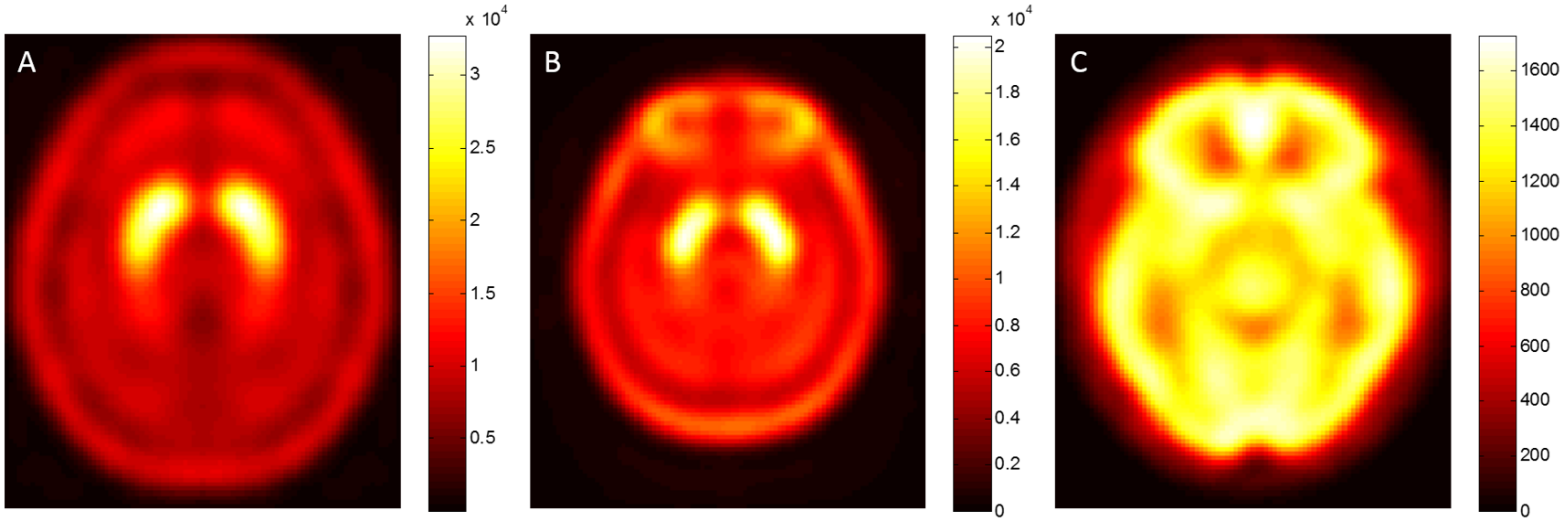
The three different templates used in this study. (A) MBT template (B) HBT template (C) HMPAO template provided in SPM8.

The most important factor that affects the accuracy of automatic image analysis is the usage of standard drug templates. However, in functional nuclear medicine imaging, different mechanisms of different brain imaging reagents or probes result in differences in accumulation of drugs in various brain regions. Therefore, the image distribution will be different and the image intensity will also be different [20]. Currently, the SPECT image template provided in the SPM software is the ^99m^Tc-HMPAO cerebral blood perfusion template. Because the distribution and pharmacokinetic characteristics of ^99m^Tc-HMPAO show large differences with those of ^99m^Tc-TRODAT-1, this will inevitably affect the accuracy of subsequent semiquantitative analysis; therefore, a template that is exclusive for ^99m^Tc-TRODAT-1 is considerably important. However, there is no template that is exclusive to ^99m^Tc-TRODAT-1 currently. Therefore, before performing automatic analysis, we must first use normal ^99m^Tc-TRODAT-1 images to create study specific templates. There are many methods for generating templates. In this study, two different templates were first generated according to different methods, which were the HMPAO-based TRODAT (HBT) template that uses the built-in ^99m^Tc-HMPAO template in SPM as the basis and the MRI-based TRODAT (MBT) template that uses MRI images as the basis [20, 21]. These two templates were used with the ^99m^Tc-HMPAO provided in the publicly available SPM8 suite for accuracy comparison (Fig 2).

#### HMPAO-based TRODAT template

The ^99m^Tc-HMPAO template is a built-in SPECT image template in SPM8. We used the “Normalise: Estimate and Write” module in SPM8 to perform spatial normalization of the ^99m^Tc-TRODAT-1 images of the 58 healthy subjects using the ^99m^Tc-HMPAO template. The parameters for spatial normalization were the same as those mentioned above (Template Image: ^99m^Tc-HMPAO template; Source Image: ^99m^Tc-TRODAT-1; Images to Write: ^99m^Tc-TRODAT-1). All ^99m^Tc-TRODAT-1 images that had undergone spatial normalization were then averaged and the images obtained at this point are preliminary HBT (pHBT) templates. Afterward, to increase and ensure the accuracy of spatial alignment, the above actions were repeated and all ^99m^Tc-TRODAT-1 images underwent spatial normalization again, but the normalization template was changed to pHBT (Template Image: pHBT template; Source Image: ^99m^Tc-TRODAT-1; Images to Write: ^99m^Tc-TRODAT-1). Finally, the ^99m^Tc-TRODAT-1 images that had undergone spatial normalization were averaged and an isotropic Gaussian kernel smoothing with 8-mm full-width-at-half-maximum (FWHM) was used [5, 10], and the HBT template was generated [10, 20].

#### MRI-based TRODAT template

Among all the healthy subjects, 7 subjects underwent both MRI T1-weighted and ^99m^Tc-TRODAT-1 SPECT imaging. First, the default settings in the “Coregister: Estimate” module in SPM8 was used for the registration of the MRI T1 images and ^99m^Tc-TRODAT-1 images of these 7 healthy subjects so that the spatial anatomical coordinates of the two conform to each other (Reference Image: MRI T1; Source Image: ^99m^Tc-TRODAT-1). Following that, the “Normalise: Estimate” module in SPM8 was used for spatial normalization of the MRI T1 images of the subjects using the MRI T1 template that was built-in in SPM8 (Template Image: MRI T1 template; Source Image: MRI T1). The normalization parameters were the same as those mentioned above. Then, the transformation parameters (*_sn.mat) produced from the “Normalise: Estimate” module was written into the corresponding registered ^99m^Tc-TRODAT-1 image by using “Normalise: Write” module (parameter file: transformation parameters; Images to Write: registered ^99m^Tc-TRODAT-1). All registered ^99m^Tc-TRODAT-1 images were averaged after this indirect spatial normalization method. Finally, an 8-mm FWHM Gaussian kernel smoothing was performed [5, 10], and the creation of the MBT template was completed [10, 18].

### Semiquantitative analysis

SUR is a commonly used marker or figure of merit (FOM) for semiquantitative analysis in brain nuclear medicine imaging [7]. Through the use of the selected ROI of the image (where ROI includes the target region in which uptake of the radiotracer is high and the background region where uptake of the radioactivity is low) and calculation of the average pixel values in ROI, the marker can be used to estimate the uptake amount of radioactivity in specific regions. The calculation of SUR is the difference between the average value of the target region and the average value of the background region divided by the average value of the background region [15]. Based on previous research [4], we choose occipital lobe as background region. Within a certain range, the higher SUR, the higher the uptake activity of this region relative to the background, as is represented by the following formula:

$$SUR\;=\;\frac{Target-Background}{Background},$$ (1)

where *Target* represents the average value of the target region ROI (striatum), and *Background* represents the average value of the background region ROI (occipital lobe region).

### Validation of phantom data

**Fig 3:**
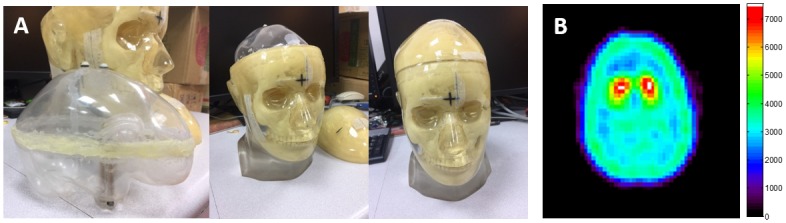
(A) Striatal phantom (B) ^99m^Tc-TRODAT-1 image of the sum of the three slices of greatest activity in striatal phantom.

This study uses striatal phantoms to simulate the ^99m^Tc-TRODAT-1 images of patients (Fig 3) [6]. The left and right sides of the phantom each contains one caudate nucleus and one putamen cavity. The caudate nuclei in both sides of the phantom were injected with three times the background activity level, the right putamen was injected with twice the background activity level and the left putamen was injected with the background activity level. The parameters used for SPECT scanning of the phantom were the same as the parameters used in the abovementioned subjects. After scanning, automatic ROI selection analysis (using the three different templates) and manual ROI selection analysis was performed. The aim of phantom testing is to validate and evaluate the feasibility and accuracy of ^99m^Tc-TRODAT-1 images in automatic semiquantitative analysis. In this study, percent error was used to compare the differences in striatal SUR between automatic and manual analytical methods and the calculation of percent error is as follows:

$$Percent\;error\;=\;\frac{\left|{SUR}_{manual}-{SUR}_{automatic}\right|}{{SUR}_{manual}}\;\times \;100,$$ (2)

where *SUR*_*manual*_ represents SUR calculated from manual ROI selection method whereas *SUR*_*automatic*_ represents SUR calculated from automatic ROI selection method.

### Validation of clinical data

After all clinical ^99m^Tc-TRODAT-1 images have undergone automatic and manual ROI selection and image analysis, SUR values of the striatum were obtained. The Pearson’s correlation coefficient of the two different methods was used to compare the correlation between automatic analysis using different templates and manual analysis. Apart from correlation, the agreement on the results between two methods was performed at the same time by drawing the Bland–Altman difference plot, calculating the variability [21] and intraclass correlation coefficient (ICC) [21, 22]. The formula for variability was:

$$Variability\;=\;\frac{\left|{SUR}_{manual}-{SUR}_{automatic}\right|}{({SUR}_{manual}+{SUR}_{automatic})/2}$$ (3)

and the formula of ICC was:

$$ICC\;=\;\frac{MSBS-MSWS}{MSBS+(k-1)MSWS}$$ (4)

where *MSBS* represents mean sum of squares between subjects, *MSWS* represents mean sum of squares within subjects, *k* represents number of within subjects measurements (k = 2 in this study, i.e. automatic and manual ROI selection measurements).

## Results

### Validation of phantom data

**Fig 4:**
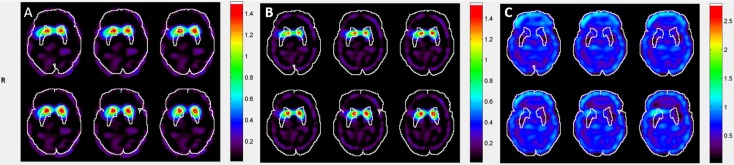
Different transverse slice of SUR maps of the striatal phantom when different templates were used for automatic ROI selection and image analysis (A) MBT template (B) HBT template (C) SPM built-in HMPAO template.

Fig 4 shows the different transverse slice of SUR maps when different templates were used for automatic ROI analysis of the striatal phantom. Table 1 shows SUR values and the percent errors of the calculated SUR values. The SUR values of the Manual, MBT, HBT, and HMPAO methods were 0.77, 0.75, 0.8, and −0.07, respectively. The percent error between MBT and Manual was the lowest at 1.61%, followed by the percent error between HBT and Manual at 4.80%, whereas the percent error between HMPAO and Manual was the greatest at 109.09%.

**Table 1:** SUR and percent error (%Error).

	SUR	%Error
**Manual**	0.77	-
**MBT**	0.75	1.61%
**HBT**	0.80	4.80%
**HMPAO**	-0.07	109.09%

### Validation of clinical data

**Fig 5:**
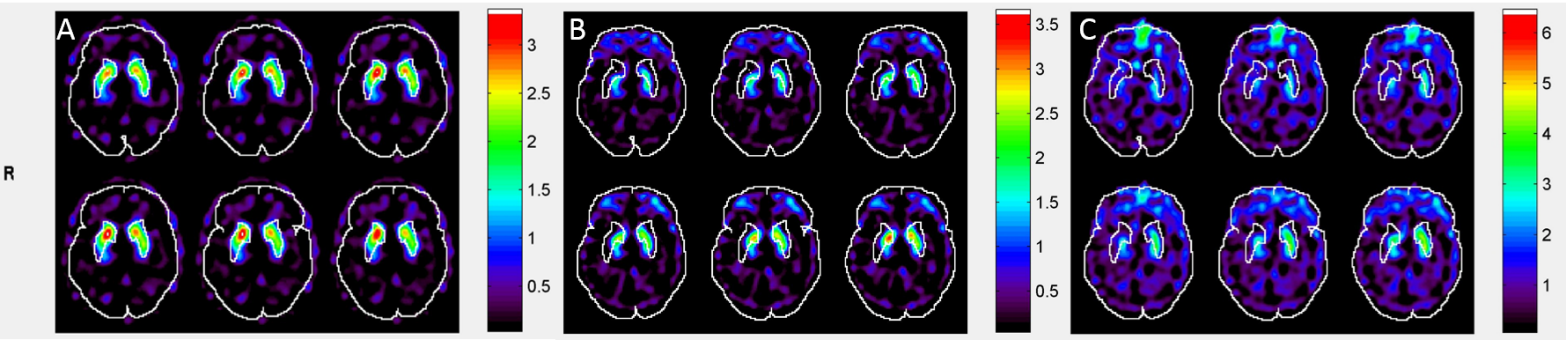
Different transverse slice of SUR maps of clinical images when different templates were used for automatic ROI selection and image analysis (A) MBT template (B) HBT template (C) SPM built-in HMPAO template.

**Fig 6:**
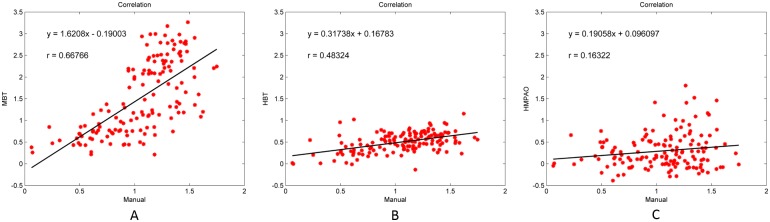
Distribution graph of SUR values from manual ROI selection method and automatic ROI selection method using different templates on clinical images. (A) Manual vs. MBT template (B) Manual vs. HBT template (C) Manual vs. HMPAO template.

**Fig 7:**
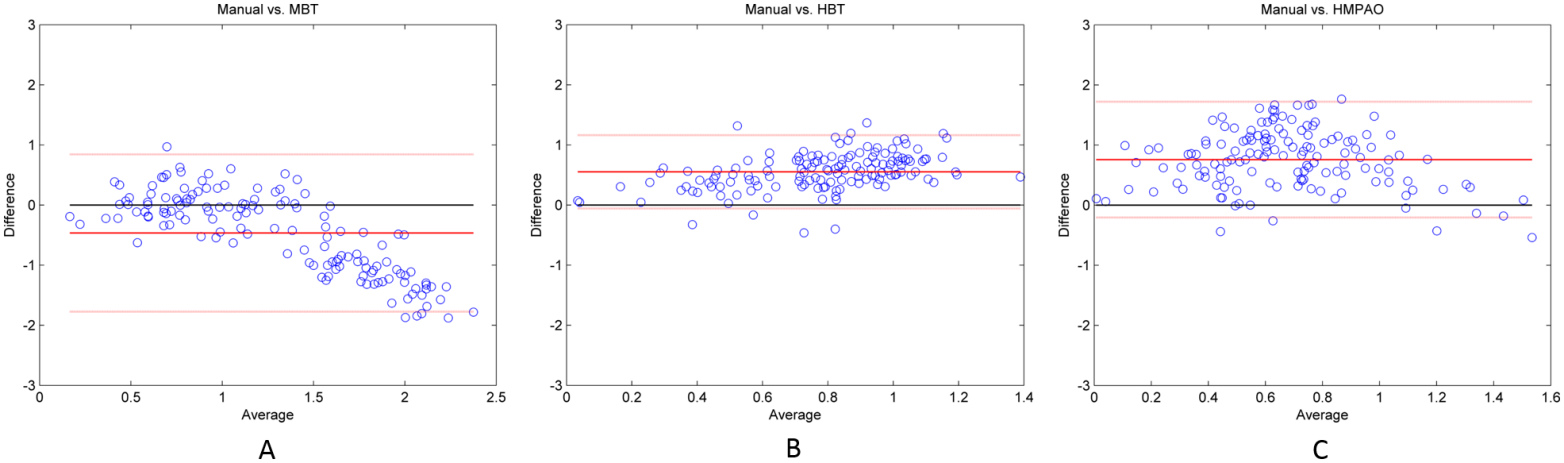
Bland–Altman difference plot of SUR values from manual ROI selection method and automatic ROI selection method using different templates on clinical images. (A) Manual vs. MBT template (B) Manual vs. HBT template (C) Manual vs. HMPAO template.

Fig 5 shows the clinical ^99m^Tc-TRODAT-1 SUR maps of different transverse slices when different templates were used for automatic ROI selection. We can see that the MBT template has the highest positional accuracy for AAL automatic ROI selection, followed by the HBT template; the HMPAO template had the worst accuracy. Fig 6 shows the correlation results of SUR values between manual analysis and automatic analysis. The SUR value calculated from the MBT template has the highest correlation with the SUR value from manual selection, with a Pearson correlation coefficient of 0.66, followed by HBT template at 0.48, and the lowest was the HMPAO template at 0.16. With regard to variability, the variability between the analysis results from the MBT template and manual selection were the lowest value at 0.44 ± 0.29, whereas the variabilities of the analysis comparison results between the HBT template and HMPAO template against manual selection were 0.78 ± 0.39 and 1.52 ± 1.58, respectively. Fig 7 shows the Bland–Altman difference plot of the SUR values between manual analysis and automatic analysis. The mean difference value between the SUR value calculated from the MBT template and the SUR value calculated from manual selection was the lowest at −0.46 (red solid lines in Fig 7), whereas the mean differences of the HBT and HMPAO templates were 0.55 and 0.75, respectively. In addition, the ICC value between the analysis results of the MBT template and manual analysis results were the highest at 0.30, whereas the ICC values for the comparison between the HBT and HMPAO templates and the manual analysis results were −0.22 and −0.41, respectively. All statistical data are summarized in Table 2, and all SUR values of subjects can be found in S1 Table.

**Table 2:** Correlation and agreement statistical analysis.

	Pearson’s corr.	Variability	Mean diff.	ICC value
**MBT vs. Manual**	0.66	0.44 ± 0.29	-0.46	0.30
**HBT vs. Manual**	0.48	0.78 ± 0.39	0.55	-0.22
**HMPAO vs. Manual**	0.16	1.52 ± 1.58	0.75	-0.41

Pearson’s corr: Pearson’s correlation coefficient; Mean diff: mean difference; ICC value: intraclass correlation coefficient value;

## Discussion

With regard to ^99m^Tc-TRODAT-1 automatic ROI selection and semiquantitative analysis, the selection of appropriate drug template is considerably important for the results of semiquantitative analysis. This is because the template will affect the accuracy of the standard MNI spatial location of the image after normalization, thereby affecting the accuracy of AAL ROI selection. Therefore, the use of an appropriate drug template can effectively increase the reliability and accuracy of semiquantitative data. Fang et al. improved the reliability and accuracy of an automatic semiquantitative analysis [23]. They proposed a novel method that using inverse transformation of ROIs to calculate the striatal-to-reference ratio (SRR) and proved that their method has a better diagnostic performance than SPM-based method. However, the SPM-based method they compared is different from our automatic method in the creation of drug template and ROIs. Their study aimed to evaluate the diagnostic performance between two automatic methods (their method and the SPM-based method). In our study, we compared our automatic methods and manual method to find the optimal automatic method that can solve the above mentioned problems caused by manual method while preserving similar results of semiquantitative analysis. Therefore, in our study, three drug templates were compared, of which two were self-created ^99m^Tc-TRODAT-1 drug templates, whereas another was the HMPAO SPECT template that was built-in in SPM. We also examined and compared the effects of these three templates on semiquantitative analysis of phantom and clinical images.

With regard to phantom images, the SUR values calculated using the MBT template has the smallest percent error (< 2%) when compared with the SUR value calculated using conventional manual ROI analysis. Therefore, with regard to automatic analysis, combination with MBT template has some feasibility and can effectively replace conventional manual analysis and improve the shortcomings associated with conventional methods, such as long duration, increased effort, low reproducibility, and human subjectivity. In the meantime, we have measured the time of automatic analysis. In all cases the analyses were performed on a 3.4 GHz Intel (R) Core (TM) i7 64 bit CPU, and the computation and analysis time per subject was about 10 seconds when using our software. This really indicates that the method can successfully save lots of time when compared with conventional method. It is worth noting that the SUR value calculated using the HMPAO template showed a phenomenon of being negative value. This shows that the activity of the target region that is selected by AAL ROI is lower than the activity of the background region. The reason for this is that the radiotracer uptake in the HMPAO template is different from that in the image to be analyzed, resulting in errors being produced during the process of spatial normalization of image intensity and causing inaccurate anatomical locations in the standard MNI space in the image after normalization. This causes the AAL ROI to select wrong locations, thereby resulting in erroneous results. This result once again demonstrated that the use of inappropriate drug templates has a considerable effect on the results of semiquantitative analysis. With regard to accuracy of spatial alignment, we performed two spatial normalizations to generate a HBT template and found that the accuracy of the analysis results was greatly improved as percent error was decreased from 109.09% to 4.80%. Although accuracy was significantly improved, this still cannot be compared with the results of the MBT template created from MRI anatomical information.

In clinical images, comparison with the results of conventional manual analysis found that the SUR value from MBT template analysis and calculation is the best in various indicators and this is consistent with the results from the phantoms. In correlation evaluations, the values calculated from MBT template had the highest correlation with conventional manual analysis results, and this approached strong correlation levels. Although HBT template results can effectively increase the accuracy of quantitative analysis and increase the correlation with manual analysis, the results are still not comparable to that of the MBT template. Therefore, the MBT template is the most suitable template among the three drug templates for use in automatic semiquantitative analysis. With regard to variability analysis, we similarly observed that the variability of the MBT template results is the lowest among the three templates. In addition, Student’s 2-sample t-tests were done (i.e. MBT vs. HBT, and MBT vs. HMPAO) to see if there was any significant difference in variability. We found that both of the results showed a significant difference (p-value < 0.01) under significant level equals to 0.05. In the Bland–Altman difference plot, the mean difference of the MBT template was the smallest among the three templates. The reason for this phenomenon is that the drug template and images that underwent spatial normalization with the MBT template has the most accurate anatomical locations in the standard MNI space. From Fig 5, we can see that when the MBT template was used for image normalization, the striatal region can be accurately covered by AAL ROI, whereas only some parts of the striatum are covered by AAL ROI when the HBT template and the HMPAO template were used for image normalization. These outcomes result in relatively lower SUR values when compared with conventional manual analytical methods. Therefore, the scores from the two templates were all distributed above the 0 baseline in the Bland–Altman difference plot, thereby causing an increase in mean differences. On the other hand, it is worth noting that some data points are distributed at the lower right corner in the Bland–Altman difference plot when the MBT template was used. This shows that the SUR values of healthy subjects that were measured from the MBT template were higher than that calculated from manual analysis. We infer that this may be because of the lack of anatomical images as aid during manual ROI selection, resulting in the selected ROI being too large and causing lower SUR values. Because of striatal defects in patients’ images, the striatal activity value has a lower difference with the background activity value; thus, there is less effect of underestimation in images obtained from PD patients. The ICC values can be used to estimate the agreement of measurements between different methods, and the use of the drug template will also affect ICC. The ICC value for the comparison of results from MBT template calculation and conventional manual analysis was the highest, showing that there is a best agreement between these two methods. In addition, the ICC values calculated when HBT and HMPAO templates were used were negative. This shows that MSWS is greater than MSBS, i.e. the difference between measurement methods (automatic and manual ROI selection) is greater than the difference between subjects. This suggests that the agreement between automatic analysis (when HBT and HMPAO templates were used) and conventional manual analysis is extremely poor, reaffirming that the MBT template is the most appropriate template for use in automatic semiquantitative analysis. According to the results of ICC value, the SUR values measured from automatic analysis using MBT template may not be the same as that measured from manual analysis; however, there is a good correlation between these two methods based on the Pearson’s correlation coefficient. As a result, we think it still has the diagnostic value for clinical evaluation of TRODAT-1 imaging. In addition, the reproducibility of the SUR measurement results using the automatic method based on MBT template is much higher than that of the conventional manual method. It is a more stable analysis method and can avoid the measurement uncertainty caused by operator errors. Therefore, we think it is worth recommending the use of automatic method based on MBT template in clinical evaluation.

From the above results, the results from the use of HBT template for automatic analysis are always better than that of from the HMPAO template, regardless of whether phantom images or clinical images are used. This is because the HBT template is obtained from the averaging and blurring of ^99m^Tc-TRODAT-1 images after spatial normalization using the HMPAO template and pHBT template; therefore, it still retains the distribution characteristics of its ^99m^Tc-TRODAT-1 radiotracer and the accuracy of the semiquantitative analysis results will be better than the HMPAO template. However, its results were still not better than the results when the MBT template was used. We observed that when the HBT template was generated using the HMPAO template as the basis, the anatomical constructs in the standard MNI space will be smaller than the AAL ROI template (Fig 2). Therefore, the accuracy of the values in the selected ROI will be decreased, resulting in its results being worse than those of the MBT template. This is because spatial normalization was performed by calculating the minimized mean-squared difference between the source image and the drug template, such that the spatial coordinate axes and size of voxels of the source image are consistent with that in the drug template. However, differences in the distribution characteristics of the drug templates cause the image intensity of the various brain regions to be different. This results in the outermost scalp region of the ^99m^Tc-TRODAT-1 images after normalization corresponding to the outermost brain region of the HMPAO template (i.e. outermost cerebral cortex) not the scalp. This results in smaller anatomical locations in the standard MNI space in the HBT template, and it affects the accuracy of quantitative analysis. Therefore, for construction of ^99m^Tc-TRODAT-1 specific templates, the use of MRI images is a better method. This is because MRI provides relatively more spatial anatomical information, enabling normalized images to have more accurate anatomical locations in the standard MNI space.

Although MBT template is the optimal template in the automatic method, there are also some restrictions. In cases of severe brain atrophy, even when the image is matched to a specific ^99m^Tc-TRODAT-1 template, particular attention should be required in the interpretation of target region after spatial normalization. In addition, because the MBT template is created from healthy subjects, this may affect the quantitation results for PD patients with different disease severity. However, we think it is not necessary to generate disease-severity-specific templates. Even patients with the same severity, the activity distribution in the image may be different. Therefore, disease-severity-specific templates may be impractical and very hard to be generated. We believe that there still exist some relations in the quantitation results between patients with different severity when using our tracer-specific template (MBT template). As a result, we still can use this automatic quantitation method with MBT template to generate disease severity databank for diagnosis and comparison.

## Conclusions

This study compared three different templates (MBT, HBT, and HMPAO [built-in in SPM] templates) that were combined with automatic analysis and manual analysis. Our results showed that the MBT template is the optimal ^99m^Tc-TRODAT-1 drug exclusive template. The exclusive drug template established was verified for use in automatic ROI selection for semiquantitative analysis of ^99m^Tc-TRODAT-1 SPECT images to quantify SUR of PD images, and this could improve the problems associated with conventional manual selection methods such as long duration, increased effort, low reproducibility, and human subjectivity. In addition, because there are few patients with both ^99m^Tc-TRODAT-1 SPECT and MRI images and even some subjects who were unable to undergo MRI imaging, therefore the use of our ready-to-use MBT templates under these circumstances could improve accuracy in semiquantitative analyses. For other agencies that wish to self-generate their own radiotracer templates, this MBT template method can be used as a basis for reference to facilitate the construction of radiotracer templates.

## Supporting information

S1 Table SUR values of all subjects. (XLSX)

## References

[1] Agid Y. Parkinson’s disease: pathophysiology. Lancet 1991; 337: 1321–1324. 1674304 10.1016/0140-6736(91)92989-f

[2] Gelb DJ, Oliver E, Gilman S. Diagnostic criteria for Parkinson disease. Arch Neurol. 1999; 56: 33–39. 9923759 10.1001/archneur.56.1.33

[3] Kung MP, Stevenson DA, Plössl K, Meegalla SK, Beckwith A, Essman WD, et al [99mTc]TRODAT-1: a novel technetium-99m complex as dopamine transporter imaging agent. Eur J Nucl Med 1997; 24: 372–380. 9096087 10.1007/BF00881808

[4] Huang WS, Lee MS, Lin JC, Chen CY, Yang YW, Lin SZ, et al Usefulness of brain 99mTc-TRODAT-1 SPET for the evaluation of Parkinson’s disease. Eur J Nucl Med. 2004; 31: 155–161 10.1007/s00259-003-1331-x 15129696

[5] Tzen KY, Lu CS, Yen TC, Wey SP, Ting G. Differential Diagnosis of Parkinson’s Disease and Vascular Parkinsonism by 99mTc-TRODAT-1. J Nucl Med. 2001; 42: 408–413 11337515

[6] Mirzaei S, Zakavi R, Rodrigues M, Schwarzgruber T, Brücke T, Bakala J, et al Fully automated 3D basal ganglia activity measurement in dopamine transporter scintigraphy (Spectalyzer). ANM. 2010; 24(4): 295–300. 10.1007/s12149-010-0353-2 20232177

[7] Buchert R, Berding G, Wilke F, Martin B, von Borczyskowski D, Mester J, et al IBZM tool: a fully automated expert system for the evaluation of IBZM SPECT studies. EJNM. 2006; 33(9): 1073–83. 10.1007/s00259-006-0067-9 16614812

[8] Fleming JS, Bolt L, Stratford JS, Kemp PM. The specific uptake size index for quantifying radiopharmaceutical uptake. Phys Med Biol. 2004; 49: 227–234. 15357202 10.1088/0031-9155/49/14/n03

[9] Costa DC, Walker Z, Dizdarevic S, Ionnides C. Striatal binding of FP-CIT: a simple method to separate Parkinson’s disease patients and normal controls. Eur J Nucl Med. 1989; 25: 1069.

[10] Lokkegaard A, Werdelin L, Friberg L. Clinical impact of diagnostic SPET investigations with a dopamine re-uptake ligand. Eur J Nucl Med. 2002; 29: 1623–1629. 10.1007/s00259-002-0938-7 12458397

[11] Morton RJ, Guy MJ, Marshall CA, Clarke EA, Hinton PJ. Variation of DaTSCAN quantification between different gamma camera types. Nucl Med Commun. 2005; 26(12): 1131–7. 16264362 10.1097/00006231-200512000-00014

[12] Morton RJ, Guy MJ, Clauss R, Hinton PJ, Marshall CA, Clarke EA. Comparison of different methods of DatSCAN quantification. Nucl Med Commun. 2005; 26(12): 1139–46. 16264363 10.1097/00006231-200512000-00015

[13] Kao PF, Tzen KY, Yen TC, Lu CS, Weng YH, Wey SP, et al The optimal imaging time for [99mTc]TRODAT-1/SPET in normal subjects and patients with Parkinson’s disease. Nucl Med Commun. 2001; 22: 151–154 11258401 10.1097/00006231-200102000-00006

[14] Yang BH, Guan YX, Wu TH. Tc-99m TRODAT-1 SPECT Imaging Protocol Guideline. Annals of Nuclear Medicine and Molecular Imaging 2016; 29: 45–53.

[15] Friston KJ. Statistical parametric mapping In: Thatcher RW, Hallett M, Zeffiro T, John ER, Huerta M, eds. Functional Neuroimaging. San Diego, CA: Academic Press; 1994: 79–93.

[16] Friston KJ, Holmes AP, Worsley KJ, Poline JP, Frith CD, Frackowiak RSJ. Statistical parametric maps in functional imaging: a general linear approach. Hum Brain Mapp. 1995; 2: 189–210.

[17] Fein G, Landman B, Tran H, Barakos J, Moon K, Di Sclafani V, et al Statistical parametric mapping of brain morphology: Sensitivity is dramatically increased by using brain-extracted images as inputs. NeuroImage 2006; 30: 1187–1195 doi: 10.1016/j.neuroimage.2005.10.054 16442817 10.1016/j.neuroimage.2005.10.054 PMC1987363

[18] Ashburner J, Friston KJ. Nonlinear spatial normalization using basis functions. Hum Brain Mapp. 1999; 7: 254–266. 10408769 10.1002/(SICI)1097-0193(1999)7:4<254::AID-HBM4>3.0.CO;2-G PMC6873340

[19] Tzourio-Mazoyer N, Landeau B, Papathanassiou D, Crivello F, Etard O, Delcroix N, et al Automated Anatomical Labeling of Activations in SPM Using a Macroscopic Anatomical Parcellation of the MNI MRI Single-Subject Brain. Neuroimage 2002; 15(1): 273–289. doi: 10.1006/nimg.2001.0978 11771995 10.1006/nimg.2001.0978

[20] Gispert JD, Pascau J, Reig S, Martínez-Lázaro R, Molina V, García-Barreno P, et al Influence of the normalization template on the outcome of statistical parametric mapping of PET scans. NeuroImage 2003; 19: 601–612 12880791 10.1016/s1053-8119(03)00072-7

[21] Kas A, Payoux P, Habert MO, Malek Z, Cointepas Y, El Fakhri G, et al Validation of a Standardized Normalization Template for Statistical Parametric Mapping Analysis of 123I-FP-CIT Images. J Nucl Med. 2007; 48: 1459–1467 doi: 10.2967/jnumed.106.038646 17704252 10.2967/jnumed.106.038646

[22] McGraw KO, Wong SP. Forming Inferences About Some Intraclass Correlation Coefficients. Psychological Methods 1996; 1: 30–46

[23] Fang YHD, Chiu SC, Lu CS, Yen TC, Weng YH. Fully Automated Quantification of the Striatal Uptake Ratio of [99mTc]-TRODAT with SPECT Imaging: Evaluation of the Diagnostic Performance in Parkinson’s Disease and the Temporal Regression of Striatal Tracer Uptake. BioMed Research International 2015; 2015: 461625 10.1155/2015/461625 PMC4558437 26366413

